# Effects of Temporal Information Structure on Causal Explanations Generated by Large Language Models: Experimental Study Using Lung Cancer Incidence Data

**DOI:** 10.2196/91101

**Published:** 2026-10-26

**Authors:** Eunzi Jeong, Wonjeong Jeong, Kyoung Hee Oh, Jae Kwan Jun

**Affiliations:** 1 Cancer Knowledge & Information Center, National Cancer Control Institute National Cancer Center Goyang, Gyeonggi-do Republic of Korea; 2 Division of Cancer Early Detection, National Cancer Control Institute, National Cancer Center Goyang, Gyeonggi-do Republic of Korea

**Keywords:** causal explanation, epidemiology, large language models, public health, temporal information

## Abstract

**Background:**

Large language models (LLMs) are increasingly applied to causal explanation in public health and epidemiology. However, recent studies suggest that LLMs exhibit instability in processing temporal information, which raises concerns about their suitability for generating coherent causal explanations. Despite this, little is known about how distinct components of temporal information influence LLM-generated causal explanations in epidemiological contexts.

**Objective:**

This study aimed to examine how event ordering and the validity and presence of explicit temporal information affect LLM-generated causal explanations of long-term lung cancer incidence trends. Specifically, we evaluated their effects on evidence grounding, causal alignment, and disease specificity.

**Methods:**

We used a 2×3 factorial design manipulating event ordering (chronological vs randomized) and explicit temporal information (accurate, inaccurate, or absent). Five LLMs (GPT-5, Gemini 3.5 Flash, Claude Opus 4.8, Qwen3-32B, and Llama 3.3 70B Instruct) generated 600 causal explanations of long-term lung cancer incidence trends using Surveillance, Epidemiology, and End Results (SEER) data and PubMed-derived event statements. The effects of ordering and information were assessed using regression and factorial ANOVA.

**Results:**

Randomized ordering showed higher evidence grounding than chronological ordering (adjusted odds ratio [aOR] 2.53, 95% CI 1.89-3.37; *P*<.001). Causal alignment was higher under chronological than randomized ordering across all 3 information conditions, with larger differences in the decreasing phase. Disease specificity was higher under randomized ordering only under accurate information.

**Conclusions:**

More accurate or structured temporal information did not necessarily improve LLM-generated causal explanations. Randomized ordering was associated with greater evidence utilization, whereas chronological ordering supported causal alignment across all information conditions, particularly for decreasing trajectories. These findings suggest that temporal context should be structured not only to provide temporal information but also to support the intended characteristics of the resulting explanation.

## Introduction

Understanding why patterns of disease occurrence change over time is an important aspect of public health and epidemiological research [1]. Epidemiological explanation is inherently temporal, because exposures and contextual factors (eg, environmental and policy conditions) evolve over time [2,3], and their effects often emerge after varying lag periods [4-7]. For chronic diseases, notably cancer, long lag periods of 10-30 years necessitate careful consideration of event ordering, temporal relevance, and temporal precedence [4,8-12]. However, real-world data frequently experience incomplete temporal information, including missing timestamps, heterogeneous time formats, and misordered events [12-14].

Meanwhile, advances in large language models (LLMs) have driven growing interest in applying them to support causal interpretation across public health and epidemiology. Prior studies have, for example, explored Socratic-style dialogues with ChatGPT (OpenAI) to generate causal interpretations of the association between fine particulate matter and mortality [15], developed LLM-based systems to assist causal reasoning in health risk assessments based on epidemiological data [16], and applied LLMs to extract cause-effect relationships from clinical practice guidelines (CPGs) [17].

However, recent natural language processing (NLP) studies have consistently highlighted structural vulnerabilities of LLMs in temporal reasoning. Even minor instability in event timing can lead to breakdowns of causal links in generated explanations [18], and LLMs are highly sensitive to temporal perturbations [19,20]. Although epidemiological causal explanation is a structured process grounded in temporal progression, LLMs often struggle to maintain temporal information in a stable and coherent manner [21]. These limitations may complicate the use of LLM-based systems for epidemiological and public health explanations.

Existing studies on the temporal reasoning capabilities of LLMs have primarily focused on tasks such as event ordering reconstruction, recognition of temporal expressions, and fact-level temporal question answering [19,22-25]. While these efforts provide an important foundation for characterizing limitations of temporal reasoning in LLMs, they are largely confined to short-range or local temporal relationships. Consequently, it remains unclear how three key elements of temporal information in public health and epidemiological data, namely, (1) event ordering [11,26,27], (2) the validity of temporal expressions [28,29], and (3) the presence or absence of temporal information [30], influence the causal explanations generated by LLMs. Despite the importance of this issue for LLM-based causal interpretation, systematic quantitative evidence remains limited.

At the same time, the reliability of LLM-generated biomedical explanations remains a concern. LLMs can generate plausible but unsupported or incorrect medical information, and hallucination benchmarks have demonstrated limitations in both medical reasoning and information recall [31]. Evidence-grounding approaches such as retrieval-augmented generation (RAG) integrate relevant external biomedical knowledge into LLM generation, with recent systematic review and meta-analytic evidence demonstrating improved performance compared with baseline LLMs [32]. Nevertheless, access to relevant evidence does not by itself ensure reliable evidence integration. Biomedical evaluations have shown that although LLMs can achieve strong performance on biomedical tasks, their outputs may still contain hallucinations or missing information [33,34]. For epidemiological causal explanation, an additional challenge is that evidence must be integrated not only factually but also according to its temporal relationship with the observed disease trajectory.

Accordingly, this study examined how event ordering and the validity and presence of explicit temporal information affect LLM-generated causal explanations of long-term disease incidence trends. Specifically, we evaluated their effects on evidence grounding, causal alignment, and disease specificity. These findings help clarify the role of temporal information in LLM-based epidemiological explanations and inform the design of future explanation systems.

## Methods

### Study Overview

This experimental study aimed to evaluate how the configuration of temporal information in input data affects causal explanations generated by LLMs. We constructed event statements containing explicit year information from PubMed articles related to lung cancer. Temporal information in the event statements was then manipulated according to 3 components: event ordering, temporal validity, and temporal presence. Based on these components, 6 experimental conditions were defined.

LLMs were provided with a time series of age-adjusted lung cancer incidence rates in the United States and a list of event statements manipulated according to each experimental condition. The models were instructed to generate causal explanations for the long-term temporal pattern of lung cancer incidence. Five LLMs, including proprietary and open-weight models, performed the same task 20 times under each of the 6 conditions. Differences in the generated causal explanations across temporal information conditions were then quantitatively evaluated.

### Lung Cancer Incidence Data

Lung cancer incidence data were obtained from the National Cancer Institute’s Surveillance, Epidemiology, and End Results (SEER) Program. The cancer site was defined as Lung and Bronchus, and delay-adjusted, age-adjusted incidence data from 1975 to 2023 were used. The data included all races and ethnicities, all ages, and both sexes, with incidence rates reported per 100,000 population. For each year, the LLMs were provided with the incidence rate, its 95% CI, and the modeled rate (trend line) provided by SEER. The same incidence time-series data were used across all models and experimental conditions.

### Event Statement Dataset

To construct an event statement dataset, we collected lung cancer–related literature from PubMed using the Entrez API. The search query included terms for lung cancer (lung cancer and the MeSH term lung neoplasms) and incidence or temporal trends (incidence, trend, and trends). These were combined with terms related to smoking and tobacco, public health policies and regulations, air pollution, and environmental or occupational exposures. The search identified 7808 PubMed identifiers (PMIDs). After removing duplicate PMIDs, abstracts were retrieved for 7746 articles. We then applied regular expression matching to identify abstracts containing explicit 4-digit year expressions in the form 19XX or 20XX, resulting in 4319 abstracts.

Event statements were extracted from these abstracts using GPT-5.4-mini (gpt-5.4-mini-2026-03-17). Each event statement described a real-world event explicitly reported in the source abstract and contained an explicit 4-digit year. Extracted events included public health policies and guidelines, screening implementation, smoking- and tobacco-related changes, environmental or occupational exposure changes, behavioral changes, public health programs, and other population-level changes. Study-specific procedures, including study periods, patient recruitment, follow-up, interventions, measurements, and data collection, were excluded. Observational findings, such as incidence or mortality trends, risk estimates, survey findings, and registry findings, were also excluded. Each statement was stored with the PMID of its source abstract to preserve its link to the original literature. This extraction resulted in 1127 candidate event statements.

To reduce extraction errors and unsupported event statements, all candidate statements were separately validated using GPT-5.5 (gpt-5.5-2026-04-23). The PMID stored as metadata for each statement was used to identify the corresponding PubMed abstract. Validation was then performed according to three predefined criteria: (1) whether the event was explicitly supported by the source abstract, (2) whether its temporal information accurately matched the source, and (3) whether the statement contained any hallucinated, fabricated, or factually incorrect information. Each candidate was classified as either KEEP or EXCLUDE. Of the 1127 candidate statements, 935 were classified as KEEP. Statements without an explicit 4-digit year were also excluded, and the remaining statements were sorted chronologically. The final dataset consisted of 764 event statements.

### Temporal Information Decomposition

For the experimental manipulation, temporal information in the event statements was decomposed into 3 components: event ordering, temporal validity, and temporal presence.

Event ordering indicated whether the sequence of event statements preserved the chronological order of the events. The ordering was considered chronological when the statements were arranged according to their original event years and randomized when their sequence was randomly rearranged.

Temporal validity indicated whether the year specified in an event statement corresponded to the original year associated with that event. It was considered accurate when the original event year was preserved and inaccurate when a different year was assigned.

Temporal presence indicated whether an event statement contained explicit year information. It was considered present when explicit year information was retained and absent when it was removed.

### Experimental Conditions

Based on the 3 temporal components, 6 experimental conditions were constructed: chronological-accurate, chronological-inaccurate, chronological-absent, randomized-accurate, randomized-inaccurate, and randomized-absent. The same 764 event statements were used across all conditions, with only their temporal information modified according to the condition. The configuration of the 3 temporal components in each condition is summarized in Table 1.

**Table 1:** Experimental conditions based on the three temporal components.

Conditions	Ordering	Information	
		Validity	Presence
Chronological-accurate	Chronological	Accurate	Present
Chronological-inaccurate	Chronological	Inaccurate	Present
Chronological-absent	Chronological	—^a^	Absent
Randomized-accurate	Randomized	Accurate	Present
Randomized-inaccurate	Randomized	Inaccurate	Present
Randomized-absent	Randomized	—	Absent

^a^N/A: not applicable.

In the chronological conditions, the original sequence of event statements was maintained, whereas the sequence was randomly shuffled in the randomized conditions. The accurate conditions preserved the original years associated with the event statements. To construct the inaccurate conditions, all 4-digit years were extracted from the event statements and randomly shuffled. The shuffled years were then reassigned to the statements, disrupting the original event–year correspondence. In the absent conditions, explicit year information was removed from the event statements. A fixed random seed of 42 was used for both sequence randomization and year reassignment to ensure reproducibility. Examples illustrating how the same event statements were represented under each condition are provided in Multimedia Appendix 1.

### LLM Experiments

Five LLMs were evaluated: GPT-5, Gemini 3.5 Flash, Claude Opus 4.8, Qwen3-32B, and Llama 3.3 70B Instruct. The first 3 were proprietary models, whereas Qwen3-32B and Llama 3.3 70B Instruct were open-weight models. Detailed model and generation settings are provided in Multimedia Appendix 2. Each model was provided with the US age-adjusted lung cancer incidence time series from 1975 to 2023 and the 764 event statements manipulated according to each experimental condition.

The models first divided the incidence time series into major temporal phases. The phases were required to be contiguous, nonoverlapping, and to cover the entire time series. They then characterized each phase as increasing or decreasing. A total of 5 event statements were selected to explain the incidence trends across the identified phases. For each phase, relevant statements among the 5 selected events were used to generate a causal explanation. Only the provided event statements could be used, and no additional events were allowed.

Outputs were returned in a structured JSON format containing the 5 selected events and the identified phases. The output included the start year, end year, trend direction, supporting events, and causal explanation for each phase. The full prompt used for causal explanation generation is provided in Multimedia Appendix 3. The task was repeated 20 times per condition across the 5 models, resulting in 600 outputs in total. As a fixed-phase sensitivity analysis, we regenerated all 600 responses using a common 3-phase structure (1975-1992, 1993-2019, and 2020-2023) across all models and conditions. The predefined boundaries were selected based on the phase structures observed in the primary experiment.

### Evaluation

Generated causal explanations were evaluated along 3 dimensions: evidence grounding, causal alignment, and disease specificity. GPT-5.6-terra was used as the evaluator for all 3 metrics and was instructed to provide a brief rationale for each evaluation.

Evidence grounding assessed whether the event statements selected by the model were actually used as evidence in the generated causal explanations. A selected statement was considered used if it was explicitly mentioned, semantically paraphrased, or its factual content was used in at least 1 phase explanation. Statements that were merely relevant to the topic or could potentially support the explanation were not considered used. Each statement was counted only once, even if it was used in multiple phases. The final score was calculated as the number of the 5 selected event statements that were used in the generated explanation, ranging from 0 to 5.

Causal alignment assessed the extent to which the causal explanation generated for each temporal phase accounted for the observed direction of change in incidence. Each phase explanation was scored from 0 to 2. A score of 2 was assigned when the explanation provided a clear and coherent causal mechanism that accounted for the observed trend, a score of 1 when the explanation was consistent with the observed trend but the causal connection was implicit or incomplete, and a score of 0 when the explanation contradicted or failed to account for the observed trend. The final score was calculated as the mean across all phases, ranging from 0 to 2.

Disease specificity assessed the extent to which each phase explanation incorporated lung cancer–specific factors or mechanisms in accounting for the observed trend. Each phase explanation was scored from 0 to 2. A score of 2 was assigned when such factors or mechanisms clearly contributed to explaining the observed trend, a score of 1 when they were relevant but their role was secondary, incomplete, or weakly connected to the trend, and a score of 0 when the explanation relied only on generic content applicable to multiple cancers or diseases. The final score was calculated as the mean across all phases, ranging from 0 to 2.

To assess the validity of the LLM-based evaluation, 120 responses (20% of the 600 generated responses) were randomly selected for manual review, with 4 responses selected from the 20 responses in each model–condition combination. The manual review was conducted by a researcher with experience in cancer and health information research. The human reviewer independently evaluated evidence grounding, causal alignment, and disease specificity using the same scoring criteria as the LLM evaluator and was blinded to the model, experimental condition, and LLM-generated scores. Agreement was assessed using exact agreement and the mean absolute score difference between the human and LLM ratings.

### Statistical Analysis

The evaluation metrics were analyzed independently. Mean scores and 95% CI were calculated across the 20 repeated runs for each model and condition. The generated response was used as the primary unit of analysis because the outcomes were defined at the response level and repeated generations were intended to capture output variability within each experimental condition.

Evidence grounding was analyzed using logistic regression, with ordering and information as predictors and model identity included to adjust for differences among models. Adjusted odds ratios (aORs) and 95% CIs were reported, and the ordering×information interaction was assessed using a Wald chi-square test. A significant ordering×information interaction was followed by simple-effects analyses comparing ordering within each information condition, with *P* values adjusted using the Holm method. To further examine the ordering effect, we calculated the utilization rate of the 5 model-selected event statements according to their positions (1-5) within the selected set.

Causal alignment and disease specificity were analyzed using factorial ANOVA, with ordering, information, and their interaction as factors and model identity included to adjust for differences among models. Effect sizes were reported as partial eta squared (partial η²). Pairwise comparisons were performed for information, and significant ordering × information interactions were followed by simple-effects analyses comparing ordering within each information condition. *P* values for multiple comparisons were adjusted using the Holm method. Additional phase-level analyses examined whether the ordering effect differed by incidence trajectory direction (increasing vs decreasing). Within each information condition and trajectory direction, ordering differences were estimated using linear regression, adjusting for model identity. Response-level cluster-robust standard errors were used to account for correlations among phases from the same response. Model-adjusted mean differences (randomized − chronological) and 95% CIs were reported. The proportions of phases with low scores (0 or 1) were also calculated by condition. For disease specificity, the model-level distribution of low disease specificity cases was additionally examined. Based on the observed model-level distribution, a sensitivity analysis was subsequently conducted using the same phase-level regression procedure. The responses generated under the fixed-phase specification were evaluated and analyzed using the same evaluation procedures and statistical models as in the primary analysis. Statistical significance was set at *P*<.05. All analyses were performed using Python with *SciPy* (version 1.17.1) and *statsmodels* (version 0.14.6).

### Ethical Considerations

Ethics approval was not sought because this study did not constitute human participants research under Article 2 of the Enforcement Rule of the Bioethics and Safety Act of the Republic of Korea. The study involved no physical intervention or interaction with human participants and did not use directly or indirectly identifiable information. Only publicly available, nonidentifiable data from the SEER Program and publicly available literature from PubMed were used. Therefore, institutional review board review and informed consent were not applicable.

## Results

Across the 600 generations, 526 (87.7%) were unique complete outputs. GPT-5, Gemini 3.5 Flash, Claude Opus 4.8, and Qwen3-32B generated 20 unique outputs in each of the 6 conditions. Llama 3.3 70B Instruct showed greater repetition, with 1 to 13 unique outputs across the 20 repeated generations depending on the condition. The number of identified phases varied across responses, with 3 phases being the most common (344/600, 57.3%), followed by 2 (145/600, 24.2%) and 4 phases (106/600, 17.7%). The distribution also varied across models and conditions. The major increasing-to-decreasing transition occurred between 1992 and 1993 in 560 (93.3%) responses. The most frequently identified additional boundary after 1993 was 2019-2020, occurring in 240 (40%) responses. Figure 1 summarizes the mean evidence grounding, causal alignment, and disease specificity scores across the 6 experimental conditions for each model.

**Figure 1:**
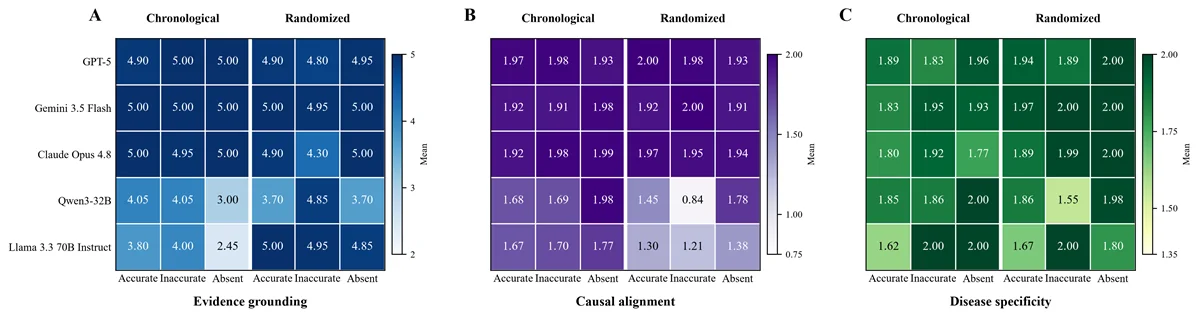
Evaluation scores across experimental conditions. Mean scores from 20 repeated runs are shown for (A) evidence grounding, (B) causal alignment, and (C) disease specificity. Columns represent 6 temporal conditions defined by the combination of temporal ordering (chronological or randomized) and information condition (accurate, inaccurate, or absent), and rows represent the evaluated large language models. Cell values indicate mean scores, with darker colors representing higher scores.

For evidence grounding, randomized ordering showed significantly higher odds than chronological ordering (aOR 2.53, 95% CI 1.89-3.37). Pairwise comparisons across information conditions showed that the absent condition had significantly lower odds than both the accurate and inaccurate conditions, whereas no significant difference was observed between the inaccurate and accurate conditions (Table 2). The ordering × information interaction was significant (Wald *χ*²_2_=8.93; *P*=.01). Post hoc simple-effects analyses showed significantly higher evidence grounding under randomized than chronological ordering only in the absent condition (aOR 4.72, 95% CI 2.93-7.62; *P*<.001), but not in the accurate or inaccurate conditions. To further examine the distribution of the ordering effect on evidence grounding, utilization rates were analyzed by position for the 5 event statements selected by the model. Under chronological ordering, the utilization rates for positions 1-5 were 100%, 92.7%, 96.3%, 79%, and 73.3%, respectively. Under randomized ordering, they were 100%, 99%, 89.7%, 89.7%, and 94%, respectively (Figure 2A).

For causal alignment, the main effects of both ordering and information were significant (*P*<.001), and the ordering×information interaction was also significant (*F*_2,590_=4.19 ηp²=0.014; *P*=.02; Table 3). Post hoc simple-effects analyses showed that causal alignment was significantly higher under chronological than randomized ordering under all 3 information conditions (Table 4). Phase-level analyses showed no significant model-adjusted mean differences (randomized − chronological) in the increasing phase. The differences were 0.046 (95% CI −0.019 to 0.110) for accurate, 0.027 (95% CI −0.045 to 0.099) for inaccurate, and −0.066 (95% CI −0.132 to 0.001) for absent. In the decreasing phase, significant model-adjusted mean differences were observed for all 3 information conditions. The differences were −0.174 (95% CI −0.263 to −0.085) for accurate, −0.487 (95% CI −0.623 to −0.350) for inaccurate, and −0.171 (95% CI −0.237 to −0.106) for absent (Figure 2B). In the decreasing phase, the proportions of low causal alignment scores (0 or 1) under chronological and randomized conditions were 17.8% and 24.9% for accurate, 11.3% and 42.1% for inaccurate, and 9.2% and 21.3% for absent, respectively.

**Table 2:** Logistic regression results for evidence grounding.

Effects		aOR^a^ (95% CI)
Ordering		
	Randomized vs chronological	2.53 (1.89-3.37)
Information		
	Absent vs accurate	0.54 (0.39-0.75)
	Inaccurate vs accurate	1.23 (0.85-1.78)
	Inaccurate vs absent	2.28 (1.62-3.21)

^a^aOR: adjusted odds ratio.

**Figure 2:**
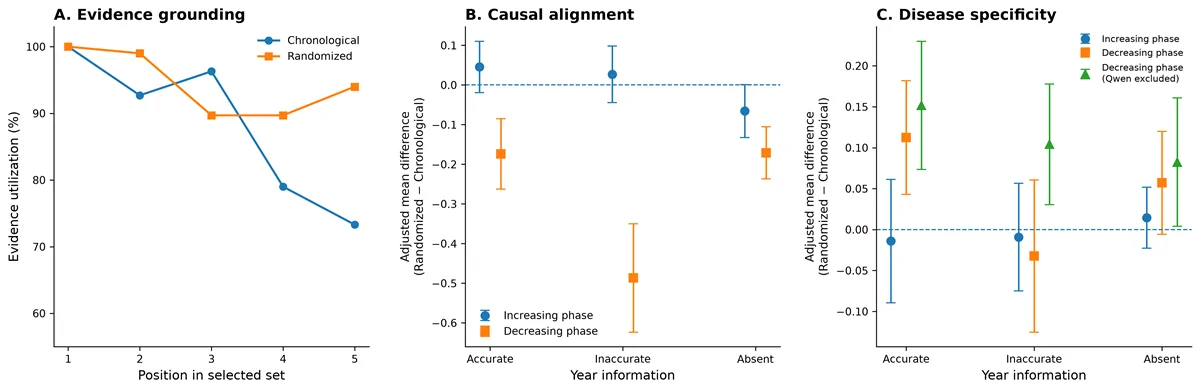
Additional analyses of large language model behavior across temporal information conditions. (A) Position-level utilization rates of the 5 event statements selected by the model, shown according to positions within the selected set under chronological and randomized. (B) Phase-level model-adjusted mean differences in causal alignment between randomized and chronological (randomized − chronological) across information conditions and incidence trajectory directions. (C) Phase-level model-adjusted mean differences in disease specificity, including the sensitivity analysis excluding Qwen for decreasing phases. Positive values indicate higher scores under randomized, whereas negative values indicate higher scores under chronological.

For disease specificity, the main effect of ordering was not significant, whereas the main effect of information and the ordering×information interaction were significant (*F*_2,590_=3.12, ηp²=0.010; *P*=.045; Table 3). Post hoc simple-effects analyses showed that disease specificity was significantly higher under randomized than chronological ordering for accurate information, whereas no significant differences were observed between the 2 ordering conditions for inaccurate or absent information (Table 4). Phase-level analyses showed no significant model-adjusted mean differences (randomized − chronological) in the increasing phase. The differences were −0.014 (95% CI −0.089 to 0.061) for accurate, −0.009 (95% CI −0.075 to 0.056) for inaccurate, and 0.014 (95% CI −0.023 to 0.052) for absent. In the decreasing phase, significant ordering differences were observed for accurate information, with a mean difference of 0.112 (95% CI 0.043-0.182). No significant differences were observed for inaccurate (mean difference −0.032, 95% CI −0.125 to 0.061) and absent information (mean difference 0.057, 95% CI −0.006 to 0.120; Figure 2C). In the decreasing phase, the proportions of low disease specificity scores (0-1) under chronological and randomized conditions were 24.2% and 16.8% for accurate, 14.5% and 20.2% for inaccurate, and 9.3% and 7.3% for absent. Further examination revealed a model-specific pattern in the randomized-inaccurate condition. Of the 36 decreasing-phase cases with low disease specificity scores, 33 were observed in Qwen. Accordingly, an exploratory post hoc sensitivity analysis excluding Qwen was conducted. In the decreasing phase, disease specificity was significantly higher under randomized conditions across all 3 information conditions. The model-adjusted mean differences (randomized − chronological) were 0.152 (95% CI 0.073-0.230) for accurate, 0.104 (95% CI 0.030-0.178) for inaccurate, and 0.082 (95% CI 0.004-0.161) for absent (Figure 2C). After excluding Qwen, the proportions of cases with low disease specificity scores in the decreasing phase under inaccurate information were 10.9% for chronological and 2.4% for randomized ordering.

In the fixed-phase sensitivity analysis, the overall ordering effects for evidence grounding and causal alignment were reproduced. Randomized ordering remained associated with higher evidence grounding (aOR 1.89, 95% CI 1.42-2.51; *P*<.001), whereas chronological ordering remained associated with higher causal alignment (*F*_1,590_=35.64, ηp²=0.057; *P*<.001). However, several findings were not reproduced under the fixed-phase specification. The information effects for evidence grounding, causal alignment, and disease specificity were no longer significant, and the ordering×information interactions were also not significant for any of the 3 outcomes.

Manual review showed exact agreement between the human and LLM evaluator for 111 of 120 responses for evidence grounding and 84 of 120 responses each for causal alignment and disease specificity. The mean absolute score differences were 0.075, 0.148, and 0.099, respectively. Among responses with different scores, the maximum differences were 1.00 point for evidence grounding and causal alignment and 0.50 points for disease specificity.

**Table 3:** Factorial ANOVA results for causal alignment and disease specificity.

Outcomes and effects			*F* test (*df*=2, 590)		*P* value
Causal alignment					
	Ordering	53.34		<.001	
	Information	11.58		<.001	
	Ordering×information	4.19		.02	
Disease specificity					
	Ordering	1.99		.16	
	Information	19.86		<.001	
	Ordering×information	3.12		.045	

**Table 4:** Simple-effects results for causal alignment and disease specificity.

Outcomes and information			MD^a^ (R^b^ − C^c^)^d^		*P* value^e^
Causal alignment					
	Accurate	−0.103		.009	
	Inaccurate	−0.259		<.001	
	Absent	−0.140		.001	
Disease specificity					
	Accurate	0.063		.04	
	Inaccurate	−0.026		.61	
	Absent	0.024		.61	

^a^MD: mean difference.

^b^R: randomized.

^c^C: chronological.

^d^Positive MDs indicate higher scores under randomized, whereas negative MDs indicate higher scores under chronological.

^e^*P* values were Holm-adjusted.

## Discussion

### Principal Findings

This study evaluated how the configuration of temporal information affects LLM-generated causal explanations of long-term lung cancer incidence trends. By independently manipulating the chronological ordering of events and the accuracy of explicit year information, we found that providing more accurate and structured temporal information did not necessarily lead to better explanations. Causal explanations might be expected to achieve the highest quality when events are assigned accurate years and presented in their actual chronological order. Previous research on temporal information has similarly identified temporal information, including time expressions, event-time relations, and event ordering, as important components [19], and has shown that chronological event representations and explicit temporal relations can improve model performance on temporal tasks [23]. However, our findings indicate that these temporal features do not necessarily translate into better overall explanation quality.

For evidence grounding, the absent condition showed significantly lower grounding than both the accurate and inaccurate conditions, whereas no significant difference was observed between the accurate and inaccurate conditions. Previous research on temporal information has treated time expressions as fundamental information for placing events within a temporal context and establishing event-time relations [19]. From this perspective, explicit years may provide temporal anchors for individual events, potentially facilitating their integration into the explanation. However, the similar levels of grounding observed under accurate and inaccurate conditions suggest that this effect did not depend on the accuracy of the temporal information itself. Randomized ordering showed higher evidence grounding than chronological ordering, and this effect of ordering varied across information conditions. Simple-effects analyses showed that the randomized advantage was significant only under the absent condition. The additional position-level analysis indicated that this difference was not uniformly distributed across positions within the selected set. Under chronological ordering, evidence utilization was high at positions 1-3 (92.7%-100%) but decreased to 79% and 73.3% at positions 4 and 5, respectively. In contrast, under randomized ordering, utilization remained between 89.7% and 100% across all positions, with 94% utilization at position 5. The higher overall evidence grounding under randomized ordering was accompanied by relatively consistent evidence utilization across positions within the selected set, whereas chronological ordering showed lower utilization at later positions. These findings suggest that in the absence of explicit temporal information, randomized ordering may not only increase overall evidence grounding but also influence how the selected evidence is used in the final explanation.

For causal alignment, chronological ordering showed higher scores than randomized ordering across all 3 information conditions, including the accurate condition. The estimated advantage of chronological ordering was larger under absent than under accurate and was largest under inaccurate. When explicit year information was absent, event sequence could provide an additional cue to the relative temporal structure of the evidence. The particularly large difference under the inaccurate condition suggests a stronger association between event ordering and causal alignment when explicit temporal information conflicts with the underlying event sequence. The difference observed under the accurate condition indicates that event ordering remained associated with causal alignment even when correct year information was explicitly provided. These findings indicate that explicit temporal anchors and chronological event ordering provide distinct temporal cues, and that the contribution of event ordering may vary according to the reliability of explicit temporal information. Phase-level analyses showed no clear differences between the 2 ordering conditions in the increasing phase, whereas causal alignment was consistently higher under chronological ordering in the decreasing phase. Qualitative examination of low causal alignment cases showed that some models attributed incidence declines to events that did not adequately explain the observed decline, including those related to screening, early detection, or mortality reduction. Chronological sequence may therefore support appropriate causal linkages between events and incidence changes beyond simply representing the order of events. Previous research has also considered chronological sequence an important temporal representation of event progression [24]. Our results further indicate that the role of sequence information may vary with both the availability and reliability of explicit temporal information and the direction of the incidence trajectory being explained.

For disease specificity, the effect of ordering varied across information conditions. Randomized ordering showed higher disease specificity than chronological ordering under the accurate condition, whereas no significant ordering differences were observed under the inaccurate or absent conditions. Phase-level analyses showed no significant ordering differences across information conditions in the increasing phase. In the decreasing phase, randomized ordering showed higher disease specificity than chronological ordering under the accurate condition, whereas no significant ordering differences were observed under the inaccurate or absent conditions. However, further examination of cases with low disease specificity scores in the decreasing phase showed that those under the randomized-inaccurate condition were concentrated in Qwen. In an exploratory post hoc sensitivity analysis excluding Qwen, randomized ordering showed higher disease specificity than chronological ordering across all 3 information conditions in the decreasing phase. To further explore the randomized advantage under the accurate condition, we qualitatively examined cases from both ordering conditions. Some chronological explanations focused on factors less specific to lung cancer, such as air pollution or broader health care factors, whereas some randomized explanations emphasized disease-specific events such as lung cancer screening. Together, these findings suggest that when accurate temporal anchors are already available, preserving chronological sequence may not necessarily enhance the use of disease-specific evidence, and that ordering may be related to which evidence is emphasized in the final explanation.

The results from the 3 measures suggest that temporal information did not affect all aspects of LLM-generated causal explanations in the same way. Rather, temporal structure appeared to play different roles in evidence utilization and causal organization. Randomized ordering was associated with greater overall evidence utilization and, under some conditions, higher disease specificity, particularly in the decreasing phase. This suggests that even when the same evidence is provided, its ordering may influence how it is used and emphasized in the final explanation. In contrast, when linking selected evidence to the incidence trajectory, preserving chronological sequence appeared to be important across all information conditions. Overall, these results suggest that greater temporal structure does not necessarily produce better causal explanations. Instead, temporal structure may affect evidence utilization and causal organization in different ways.

This study has several limitations. First, the evaluation was based on 5 LLMs, lung cancer incidence, and a predefined set of event statements, and further studies are needed to determine whether these findings generalize to other diseases or time-series outcomes. Second, the evaluation relied on LLM-based assessment, which may be subject to evaluator-specific biases or variability despite the use of predefined scoring criteria and the additional manual review of a randomly selected subset. Third, the phase-level and low-score case analyses were post hoc and therefore should not be interpreted as direct evidence of the internal mechanisms of LLMs. Fourth, because responses were regenerated for the fixed-phase analysis, differences from the primary analysis may partly reflect stochastic variation.

### Conclusions

This study showed that providing more accurate or structured temporal information does not necessarily improve LLM-generated causal explanations. Randomized ordering was associated with greater evidence utilization, whereas chronological ordering supported causal alignment across all information conditions, particularly for decreasing trajectories. Thus, effective temporal explanation requires more than simply providing additional temporal information. These findings suggest that temporal context in LLM-based explanation systems should be designed not only to provide well-structured temporal information, but also to support the intended characteristics of the resulting explanation.

## Appendix

Multimedia Appendix 1 Examples of event statement manipulations across experimental conditions.

Multimedia Appendix 2 Large language models and generation settings used in the experiments.

Multimedia Appendix 3 Prompt for causal explanation generation.

## Abbreviations

aOR: adjusted odds ratio
CPG: clinical practice guideline
LLM: large language model
NLP: natural language processing
PMID: PubMed identifier
RAG: retrieval-augmented generation
SEER: Surveillance, Epidemiology, and End Results

## Data Availability

The epidemiological incidence data analyzed in this study are publicly available from the Surveillance, Epidemiology, and End Results (SEER) Program of the US National Cancer Institute. Textual data used for causal explanation generation were obtained from publicly accessible PubMed abstracts via the Entrez Programming Utilities (E-utilities) API. All data sources used in this study are publicly available and contain no personally identifiable information.
