# Supplementary material for: Effects of Temporal Information Structure on Causal Explanations Generated by Large Language Models: Experimental Study Using Lung Cancer Incidence Data
Source: J Med Internet Res. 2026 Oct 26;28:e91101. doi: 10.2196/91101 (PMC13749024; doi:10.2196/91101)
Supplement: Supplementary file 1 [file jmir_v28i1e91101_app1.docx]

Multimedia Appendix 1 Examples of Event Statement Manipulations Across Experimental Conditions

**Chronological–Accurate**

- In 1957, the surgeon general and US Public Health Service scientists concluded that smoking was a cause of lung cancer.
- In 1988, the California tobacco control program was enacted.
- In 2013, the US Preventive Services Task Force recommended lung cancer screening with low dose computed tomography for early detection of lung cancer.

**Chronological–Inaccurate**

- In 2022, the surgeon general and US Public Health Service scientists concluded that smoking was a cause of lung cancer.
- In 1995, the California tobacco control program was enacted.
- In 2000, the US Preventive Services Task Force recommended lung cancer screening with low dose computed tomography for early detection of lung cancer.

**Chronological–Absent**

- the surgeon general and US Public Health Service scientists concluded that smoking was a cause of lung cancer.
- the California tobacco control program was enacted.
- the US Preventive Services Task Force recommended lung cancer screening with low dose computed tomography for early detection of lung cancer.

**Randomized–Accurate**

- In 1988, the California tobacco control program was enacted.
- In 2013, the US Preventive Services Task Force recommended lung cancer screening with low dose computed tomography for early detection of lung cancer.
- In 1957, the surgeon general and US Public Health Service scientists concluded that smoking was a cause of lung cancer.

**Randomized–Inaccurate**

- In 1995, the California tobacco control program was enacted.
- In 2000, the US Preventive Services Task Force recommended lung cancer screening with low dose computed tomography for early detection of lung cancer.
- In 2022, the surgeon general and US Public Health Service scientists concluded that smoking was a cause of lung cancer.

**Randomized–Absent**

- the California tobacco control program was enacted.
- the US Preventive Services Task Force recommended lung cancer screening with low dose computed tomography for early detection of lung cancer.
- the surgeon general and US Public Health Service scientists concluded that smoking was a cause of lung cancer.
