# Supplementary material for: Effects of Temporal Information Structure on Causal Explanations Generated by Large Language Models: Experimental Study Using Lung Cancer Incidence Data
Source: J Med Internet Res. 2026 Oct 26;28:e91101. doi: 10.2196/91101 (PMC13749024; doi:10.2196/91101)
Supplement: Supplementary file 2 [file jmir_v28i1e91101_app2.docx]

Multimedia Appendix 2 LLMs and Generation Settings Used in the Experiments

| **Model** | **Model ID** | **Model type** | **Generation settings** |
| --- | --- | --- | --- |
| GPT-5 | gpt-5 | Proprietary | Generation parameters were not explicitly specified in the API call |
| Gemini 3.5 Flash | gemini-3.5-flash | Proprietary | JSON response format (application/json); other generation parameters were not explicitly specified |
| Claude Opus 4.8 | claude-opus-4-8 | Proprietary | max_tokens = 8192; other generation parameters were not explicitly specified |
| Qwen3-32B | Qwen/Qwen3-32B | Open-weight | FP16; max_new_tokens = 8192; do_sample = True; thinking mode disabled |
| Llama 3.3 70B Instruct | meta-llama/Llama-3.3-70B-Instruct | Open-weight | FP16; max_new_tokens = 8192; do_sample = True |
