# Supplementary material for: Effects of Temporal Information Structure on Causal Explanations Generated by Large Language Models: Experimental Study Using Lung Cancer Incidence Data
Source: J Med Internet Res. 2026 Oct 26;28:e91101. doi: 10.2196/91101 (PMC13749024; doi:10.2196/91101)
Supplement: Supplementary file 3 [file jmir_v28i1e91101_app3.docx]

Multimedia Appendix 3 Prompt for Causal Explanation Generation

**System prompt**

You are an epidemiologist.

Your task is to generate an evidence-based causal explanation for long-term changes in lung cancer incidence in the United States.

Use only the provided evidence.

Do not introduce events that are not supported by the provided evidence.

**User prompt**

Below is the age-adjusted lung cancer incidence in the United States from 1975 to 2023.

    {seer}

    ----------------------------------------

    Below are event statements that may provide evidence for explaining the long-term temporal pattern of lung cancer incidence.

    {evidence}

    ----------------------------------------

    Task

    Using only the provided evidence, explain the long-term temporal pattern of lung cancer incidence in the United States.

    Your response should:

    1. Divide the overall incidence trend into the major phases that you identify from the incidence data.

      - The phases must be contiguous and non-overlapping.

      - Each year should belong to exactly one phase.

      - Specify the start and end year of each phase using four-digit years.

    2. Select exactly five event statements from the provided evidence that could plausibly explain the overall temporal pattern of lung cancer incidence.

      - The five event statements should be selected in total, not separately for each phase.

      - The same selected event statement may support more than one phase.

    3. For each phase:

      - Specify the overall trend as either "increasing" or "decreasing".

      - Indicate which of the five selected event statements support that phase.

      - Explain how the selected event statements account for the observed trend during that phase using only the provided evidence.

      - Write naturally. Do not explicitly refer to "the selected events", "the selected evidence", or "the provided evidence".

    Copy the selected event statements exactly as written.

    Do not paraphrase, summarize, or modify them.

    Do not include any event that is not present in the provided evidence.

    Return ONLY valid JSON.

    {{

      "selected_events": [

        "...",

        "...",

        "...",

        "...",

        "..."

      ],

      "phases": [

        {{

          "start_year": 1975,

          "end_year": 1992,

          "trend": "increasing",

          "supporting_events": [

            "...",

            "..."

          ],

          "explanation": "..."

        }},

        {{

          "start_year": 1993,

          "end_year": 2007,

          "trend": "decreasing",

          "supporting_events": [

            "...",

            "..."

          ],

          "explanation": "..."

        }}

      ]

    }}
